# Use of electrical neuromuscular stimulation to preserve the morphology of abdominal and chest muscles of critical patients: randomized clinical trial

**DOI:** 10.1186/2197-425X-3-S1-A552

**Published:** 2015-10-01

**Authors:** LJ Santos, AM Dall' Acqua, A Sachetti, FA Lemos, T Bianchi, WS Naue, G Sbruzzi, AS Dias, SR Vieira, MoVe-ICU Group

**Affiliations:** Hospital de Clínicas de Porto Alegre, Porto Alegre, Brazil

## Introduction

Neuromuscular electrical stimulation (NMES) has been used as an early therapeutic modality at intensive care units (ICUs) to treat patients on invasive mechanical ventilation (IMV) to compensate and/or decrease loss of muscle mass.

## Objective

To evaluate and compare the effects of NMES combined with conventional physical therapy on muscle thickness of critically ill patients on IMV.

## Methods

Double blind randomized controlled trial conducted at the ICU of the Hospital de Clínicas de Porto Alegre, Brazil. Twenty-five patients who had been in hospital for at most 15 days and were receiving IMV for 24 to 48 hours were included in the study. Patients were randomized to the intervention group (NMES + conventional physical therapy) or conventional group (conventional therapy + placebo NMES). Interventions were conducted daily for 30 minutes until the seventh day or upon extubation.

## Results

The primary outcome was thickness of the transverse rectus abdominis and chest muscles of the dominant side assessed by ultrasound before and after the intervention. Eleven patients were included in the intervention group (56 ± 13 years) and fourteen in the conventional group (61 ± 15 years). After NMES administration, rectus abdominis muscle thickness (0.47 ± 0.08 before vs. 0.51 ± 0.08 after, p = 0.505) and chest muscle thickness (0.44 ± 0.08 before vs. 0.49 ± 0.08 after, p = 0.083) were preserved in the intervention group, whereas there was significant reduction of thickness in the conventional group (rectus abdominis: 0.43 ± 0.05 before vs. 0.36 ± 0.04 after, p = 0.001; chest: 0.42 ± 0.05 before vs. 0.35 ± 0.04 after, p = 0.001), with a significant difference between the groups. There was statistically significant difference between the groups in terms of length of ICU stay, with shorter length of stay in the intervention group (10 ± 4, p = 0.045). We found no significant difference related to the other secondary outcomes between the groups.

## Conclusion

There was no change in the rectus abdominis and chest muscle thickness in the intervention group; however, we found a significant decrease in the measures in the conventional group.
